# The CO_2_-dependence of *Brucella ovis* and *Brucella abortus* biovars is caused by defective carbonic anhydrases

**DOI:** 10.1186/s13567-018-0583-1

**Published:** 2018-09-05

**Authors:** Lara Pérez-Etayo, María Jesús de Miguel, Raquel Conde-Álvarez, Pilar M. Muñoz, Mammar Khames, Maite Iriarte, Ignacio Moriyón, Amaia Zúñiga-Ripa

**Affiliations:** 1 Instituto de Salud Tropical y Departamento de Microbiología y Parasitología-IDISNA, Universidad de Navarra, 31008 Pamplona, Spain; 2 Unidad de Producción y Sanidad Animal del Centro de Investigación y Tecnología Agroalimentaria de Aragón (CITA), Instituto Agroalimentario de Aragón (IA2), Zaragoza, Spain; 3 Department of Biology, University of Medea, 26000 Medea, Algeria; 4 National Veterinary High School, Algiers, Algeria

## Abstract

*Brucella* bacteria cause brucellosis, a major zoonosis whose control requires efficient diagnosis and vaccines. Identification of classical *Brucella* spp. has traditionally relied on phenotypic characterization, including surface antigens and 5–10% CO_2_ necessity for growth (CO_2_-dependence), a trait of *Brucella ovis* and most *Brucella abortus* biovars 1–4 strains. Although molecular tests are replacing phenotypic methods, CO_2_-dependence remains of interest as it conditions isolation and propagation and reflects *Brucella* metabolism, an area of active research. Here, we investigated the connection of CO_2_-dependence and carbonic anhydrases (CA), the enzymes catalyzing the hydration of CO_2_ to the bicarbonate used by anaplerotic and biosynthetic carboxylases. Based on the previous demonstration that *B. suis* carries two functional CAs (CAI and CAII), we analyzed the CA sequences of CO_2_-dependent and -independent brucellae and spontaneous mutants. The comparisons strongly suggested that CAII is not functional in CO_2_-dependent *B. abortus* and *B. ovis*, and that a modified CAII sequence explains the CO_2_-independent phenotype of spontaneous mutants. Then, by mutagenesis and heterologous plasmid complementation and chromosomal insertion we proved that CAI alone is enough to support CO_2_-independent growth of *B. suis* in rich media but not of *B. abortus* in rich media or *B. suis* in minimal media. Finally, we also found that insertion of a heterologous active CAII into *B. ovis* reverted the CO_2_-dependence but did not alter its virulence in the mouse model. These results allow a better understanding of central aspects of *Brucella* metabolism and, in the case of *B. ovis*, provide tools for large-scale production of diagnostic antigens and vaccines.

**Electronic supplementary material:**

The online version of this article (10.1186/s13567-018-0583-1) contains supplementary material, which is available to authorized users.

## Introduction

*Brucella* is a genus of gram-negative bacteria of the α-2 subdivision of the class *Proteobacteria* [1] that includes the causal agents of brucellosis, a zoonosis producing important economical loses and human suffering in many developing countries [2]. Currently, the genus contains twelve nominal species often showing host preference. Those spp. that were identified early (frequently referred to as the classical *Brucella* spp.) are *Brucella abortus*, preferentially infecting cattle, *B. melitensis*, usually infecting sheep and goats, *B. suis*, infecting pigs, hares, reindeer ad several wild rodents, *B. canis*, found in dogs, *B. neotomae*, isolated from desert wood rats, and *Brucella ovis*, a non-zoonotic species that is restricted to sheep and causes a condition known as *B. ovis* ovine epididymitis [3]. More recently, brucellae have been isolated from marine mammals, voles and other wild life vertebrates, and new species proposed [4–8].

Conventional identification of the classical *Brucella* spp. and biovars has traditionally relied on dye and phage sensitivity, H_2_S production, urease activity, requirement of 5–10% CO_2_ atmospheres (0.04% in normal air) for growth (CO_2_-dependence) and surface antigens [9]. Even though these methods are being rapidly replaced by molecular tests, antigenic structure and CO_2_-dependence remain of immediate practical interest as these properties affect the conditions for primary isolation and propagation in vitro and the implementation of diagnostic tests. Antigenically, the classical *Brucella* spp. are divided in two groups: the rough (R) species (*B. canis* and *B. ovis*), which carry R-type lipopolysaccharides (LPS), and the smooth (S) brucellae, which carry S-LPS [3, 10]. Concerning CO_2_-dependence, this is a trait of *B. ovis* and most strains of *B. abortus* biovars 1–4 [3, 10]. In both cases, CO_2_-independent variants may appear with low frequency [11] and, for obvious practical reasons, some of these variants have been used for *B. ovis* and *B. abortus* antigen production [3, 10]. Similarly, *B. abortus* vaccines have been developed on CO_2_-independent backgrounds [10].

The classical *Brucella* spp. are facultative intracellular parasites able to circumvent early proinflammatory responses and endowed with a type IV secretion system involved in the control of intracellular trafficking [12–15]. Moreover, it is postulated that these bacteria have progressively adapted their metabolism to the nutrients encountered within cells as an essential part of their intracellular strategy [16–21]. In this regard, despite being a notorious phenotype of practical importance, *Brucella* CO_2_-dependence has deserved no attention since the demonstration over 60 years ago that CO_2_-rich atmospheres are not required to reduce oxygen tension and that CO_2_ is used as a nutrient per se (reviewed in [11]). Indeed, CO_2_ assimilation requires carbonic anhydrases (CAs), a group of critically important ubiquitous enzymes distributed into six evolutionary distinct classes named α to η, with the β class present in bacteria [22]. In heterotrophs, CAs are involved in C acquisition via assimilatory and anaplerotic reactions linked to several biosynthetic processes [23], and CO_2_-dependence has been related to defects in CA function in several microorganisms [24–30]. However, to the best of our knowledge, the role of CAs in CO_2_-dependence has not been investigated in *Brucella* spp. where the information is limited to recent investigations in search for targets for new drugs [31–34]. These investigations have described that *B. suis* (thus CO_2_-independent) strain 1330 has two ORFs (BRA0788 and BR1829) that code for β CAs (henceforth _Bs1330_CAI and _Bs1330_CAII). Both CAs are predicted to contain all the amino acid residues involved in the catalytic site and, more important, their activity was verified upon purification and found to be better for _Bs1330_CAII [31–33]. The demonstration that these *B. suis* ORFs actually code for enzymes with the predicted activity, together with the availability of the genome sequences of both CO_2_-independent and -dependent *Brucella* spp. and biovars, open the way to investigate the mechanisms underlying CO_2_-dependence in *Brucella.* The aim of the work described here was twofold: to investigate the genetic background behind the *Brucella* CO_2_-independent and -dependent phenotypes and, for *B. ovis*, a species that shows constant CO_2_-dependence, to construct a CO_2_-independent strain suitable for vaccine and antigen production.

## Materials and methods

### Bacterial strains and growth conditions

The bacterial strains and plasmids used in this study are listed in Table 1 and Additional files 1 and 2. *B. abortus* and *B. suis* strains were grown in standard Peptone-Glucose (Tryptic soy broth, TSB) or in this medium supplemented with agar (TSA). *B. ovis* strains were grown in TSB supplemented with yeast extract (0.5%) and fetal bovine serum 5% (TYSB) or this medium supplemented with agar (TYSA). For the studies in mice, strains were growth in Blood Agar Base supplemented with fetal bovine serum 5% (BABS). In addition, two minimal media were used. The components for 1 L of the defined medium of Gerhardt (Glutamate–Lactate–Glycerol) [35] are: glycerol (30 g), lactic acid (5 g), glutamic acid (1.5 g), thiamine (0.2 mg), nicotinic acid (0.2 mg), pantothenic acid (0.04 mg), biotin (0.0001 mg), K_2_HPO_4_ (10 g), Na_2_S_2_O_3_∙5H_2_O (0.1 g), MgSO_4_ (10 mg), MnSO_4_ (0.1 mg), FeSO_4_ (0.1 mg) and NaCl (7.5 g). The pH was adjusted to 6.8–7. The second minimal medium was a modification of Plommet’s [17, 36] and 1 L of this medium is composed of thiamine (0.2 g), nicotinic acid (0.2 g), pantothenic acid (0.07 g), biotin (0.1 mg), K_2_HPO_4_ (2.3 g), KH_2_PO_4_ (3 g), Na_2_S_2_O_3_ (0.1 g), MgSO_4_ (0.01 g), MnSO_4_ (0.1 mg), FeSO_4_ (0.1 mg); NaCl (5 g), (NH_4_)_2_SO_4_ (0.5 g) and 1 g/L of glucose. Incubation was at 37 °C, with (5%) or without CO_2_. When needed, media were supplemented with 5% sucrose (Sigma), kanamycin (Km) at 50 μg/mL, nalidixic acid (Nal) at 25 μg/mL, polymyxin (Pmx) at 1.5 μg/mL, chloramphenicol (Cm) at 20 μg/mL, spectinomycin (Spc) at 100 μg/mL or ampicillin (Amp) at 100 μg/mL (all from Sigma). All strains were stored in skimmed milk or TYSB-DMSO at −80 °C.

**Table 1:** **Characteristics of the** ***Brucella*** **strains used in CO**_**2**_ **-dependence studies**

Strain (biovar)	CO_2_-dependence	Other relevant characteristics	References (code)
Reference/collection strains			
*B. suis* 1330 (1)	–	Virulent; reference strain of biovar 1; ATCC 23444	[9]
*B. suis* 513 (5)	–	Virulent; reference strain of biovar 5; NCTC 11996	[9, 45, 61]
*B. abortus* 2308W (1)	–	Virulent; Wisconsin replicate of USDA challenge strain 2308	[45, 62]
*B. abortus* 544 (1)	+	Virulent; reference strain of biovar 1; ATCC 23448	[9]
*B. abortus* 292 (4)	+	Virulent; reference strain of biovar 4; ATCC 23451	[9]
*B. ovis* PA (n.a.)^a^	+	Virulent; challenge strain used in *B. ovis* vaccine studies.	[63–65]
*B. ovis* REO198 (n.a.)^a^	–	Attenuated; genome not sequenced; used for R antigen production for serodiagnosis of ovine epididymitis	[3, 66]
Field strains			
*B. abortus* AB0339 (1)	+	Cattle isolate	[44]
*B. abortus* AB0127 (3)	+	Cattle isolate	[44]
*B. abortus* AB0130 (3)	+	Cattle isolate	[44]
Spontaneous mutants			
BoPA-CO_2_^mut^ (n.a.)^a^	–	*B. ovis* PA mutant isolated during routine work at CITA	This work (Ov-2357)
AB0339-CO_2_^mut^ (1)	–	*B. abortus* mutant isolated during routine work at University of Navarra	This work (AZB250)
AB0127-CO_2_^mut^ (3)	–	*B. abortus* mutant isolated during routine work at University of Navarra	This work (AZB251)
AB0130-CO_2_^mut^ (3)	–	*B. abortus* mutant isolated during routine work at University of Navarra	This work (AZB252)

^a^ *n.a.* not applicable (no biovars defined for *B. ovis*).

### Sequence analyses

Genomic sequences of *B. suis* 1330, *B. suis* 513 (not annotated), *B. abortus* 544 (not annotated) and *B. abortus* 292 were obtained from the databases at National Center for Biotechnology Information (NCBI), Kyoto Encyclopedia of Genes and Genomes (KEGG) or The Broad Institute. The genomic sequence of *B. abortus* 2308W was obtained from the European Nucleotide Archive (ENA) and compared with its sibling 2308 sequence in KEGG. When genomic sequences were not available (*B. ovis* PA, *B. ovis* REO198, *B. abortus* AB0339, AB0339-CO_2_^mut^, *B. abortus* AB0127, AB0127-CO_2_^mut^, *B. abortus* AB0130, AB0130-CO_2_^mut^ and BoPA-CO_2_^mut^) ORFs were PCR amplified and then sequenced. DNA sequencing was carried out by “Servicio de Secuenciación de CIMA (Centro de Investigación Médica Aplicada, Pamplona, Spain)”. Sequence alignments were performed with Clustal Omega.

### DNA manipulations

Plasmid and chromosomal DNA were extracted with QIAprep Spin Miniprep (Qiagen) and Ultraclean Microbial DNA Isolation kit (Mo Bio Laboratories), respectively. When needed, DNA was purified from agarose gels using QIAquick Gel Extraction Kit (Qiagen). Primers (Additional file 3) were synthesized by Sigma (Haverhill, United Kingdom). Restriction modification enzymes were used under the conditions recommended by the manufacturer.

### Construction of *B. abortus* 2308W and *B. suis* mutants by gene disruption

For the construction of the CAI mutants, an internal region of 323 bp was amplified with oligonucleotides CAI-F1-ins (5′-GAATTTCTATGGATCGGCTGTT-3′) and CAI-R2-ins (5′-CGGTCCTGCGTGTTTTCTAT-3′). The resulting fragment containing an internal region of the ORF was cloned into pCR2.1-TOPO^®^ vector (Invitrogen) to generate plasmid pCR2.1_Ba2308W_CAI (Additional file 2) and then, sequenced to verify the insertion. After sequencing, this fragment was cloned into the *Bam*HI and *Xba*I sites of the suicide vector pJQKm [37]. The resulting plasmid pJQKm_Ba2308W_CAI (Additional file 2) was transformed into competent *E. coli* S17 λpir [38, 39] and transferred into *B. abortus* 2308W, *B. suis* 1330 and *B. suis* 513 by conjugation, where a single crossover led to disruption of the wild type locus. Integrative mutants were selected on a medium containing kanamycin and nalidixic acid or polymyxin and called *B. abortus* 2308W::pJQKm-CAI, *B. suis* 513::pJQKm-CAI and *B. suis* 1330::pJQKm-CAI (Additional file 1). Since the orientation of the insert in the pJQKm vector was known after sequencing, gene disruption was confirmed by detecting PCR products with primers CAI-Fw and M13Fw and primers CAI-Rv and M13Rv.

The CAII mutants were constructed in a similar way. A 302 bp internal fragment of CAII was amplified with oligonucleotides CAII-F1-Ins (5′-CAATGTGGCCAATCTCATTC-3′) and CAII-R2-ins (5′-GCGAATAGCGGATCGAAATA-3′). The resulting fragment was cloned into pCR2.1-TOPO^®^ vector (Invitrogen) to generate plasmid pCR2.1_Ba2308W_ CAII (Additional file 2), sequenced to verify the insertion and subsequently cloned into the *Bam*HI and *Xba*I sites of the suicide vector pJQKm [37]. The mutants were named *B. suis* 513::pJQKm-CAII and *B. suis* 1330::pJQKm-CAII (Additional file 1). Since the orientation of the insert in the pJQKm vector was known after sequencing, the site of the insertion was confirmed by independent PCR rounds with primers CAII-Fw and M13Fw, and primers CAII-Rv and M13Rv. After several attempts, no mutant in *B. abortus* 2308W CAII was obtained either under normal or CO_2_-enriched conditions.

### Selection of CO_2_-independent spontaneous mutants

To obtain CO_2_-independent spontaneous mutants from CO_2_-dependent bacteria, *B. ovis* PA and three *B. abortus* isolates (one biovar 1 and two biovar 3) were plated on TYSA or TSA and incubated at 37 °C without CO_2_. After 5 days, one colony was picked and the genes encoding CAI and CAII were PCR amplified using primers CAI-Fw and CAI-Rv, and CAII-Fw and CAII-Rv (see Additional file 3). DNA sequencing with these primers and CAI-F1-Sec and CAII-F1-Sec primers (Additional file 3) allowed identification of mutations by comparison with the nucleotide sequence of the parental CO_2_-dependent strains.

### Construction of the plasmid carrying CAII_ba2308W_ and introduction into *B. abortus* 292 and 544

For the construction of the expression plasmid encoding _Ba2308W_CAII oligonucleotides CAII-Fw-Gw (5′-GGGGACAAGTTTGTACAAAAAAGCAGGCTTCCGCTGCCGTGTTTGAAATCA-3′) and CAII-Rv-Gw (5′-GGGGACCACTTTGTACAAGAAAGCTGGGTCTCAAAGTTCAGGGCGTTTGAA-3′) that contain sequences *attB* (underlined) were used to amplify CAII and the promoter from *B. abortus* 2308W. The resulting PCR product was cloned into pDONR223 to generate plasmid pDONOR223_Ba2308W_CAII (Additional file 2). After sequence verification, the ORF encoding CAII was transferred from pDONOR223_Ba2308W_CAII to pRH001 [40]. The resulting plasmid, pRH001_Ba2308W_CAII (Additional file 2) was transformed into competent *E. coli* S17 λpir and introduced into *Brucella* strains by conjugation. The clones that had acquired the plasmid were selected by kanamycin resistance and confirmed by PCR using primers CAII-Fw-Gw and CAII-Rv-Gw, and M13F-M13R. The strains were called *B. abortus* 292 pRH001_Ba2308W_CAII and *B. abortus* 544 pRH001_Ba2308W_CAII (Additional file 1).

### Construction of miniTn7T-Km^R^ plasmids carrying CAI or CAII and introduction into *Brucella* strains

Using DNA from *B. abortus* 2308W, oligonucleotides CAII-IF-F1 (5′-CCGGGCTGCAGGAATTCGCTGCCGTGTTTGAAATCA-3′) and CAII-IF-R2 (5′-AGCTTCTCGAGGAATTTCAAAGTTCAGGGCGTTTGAA-3′) amplified a 966 bp region containing CAII and the promoter region. This fragment was cloned into the linearized vector (*Eco*RI) pUC18R6KT-miniTn7T-Km^R^ [41] using the In-Fusion HD Enzyme Premix (Clontech). The resulting plasmid was called pUC18R6KT-miniTn7T-Km^R^_Ba2308W_CAII (Additional file 2) and transformed into *E. coli* PIR1 and subsequently to *E. coli* S17 λpir. Then, it was transferred into *Brucella* by a tetraparental conjugation [42]. The resulting constructs (*B. abortus* 292 Tn7_Ba2308W_CAII, *B. abortus* 544 Tn7_Ba2308W_CAII and *B. ovis* PA Tn7_Ba2308W_CAII; Additional file 1) were confirmed by PCR for the correct insertion and orientation of the mini-Tn7 between genes *glmS* and *recG.* Primers GlmS_B (5′-GTCCTTATGGGAACGGACGT-3′) and Ptn7-R (5′-CACAGCATAACTGGACTGATT-3′) were used to confirm insertion downstream *glmS*; Ptn7-L (5′-ATTAGCTTACGACGCTACACCC-3′) and RecG (5′-TATATTCTGGCGAGCGATCC-3′) insertion upstream *recG* and GlmS_B and RecG presence of transposon.

Oligonucleotides CAI-IF-F1 (5′-CCGGGCTGCAGGAATTTGTGGAATTGCACCGACAC-3′) and CAI-IF-R2 (5′-AGCTTCTCGAGGAATTCAATTATTCTGCCGGTTGG-3′) amplified a 987 bp fragment from *B. suis* 513 DNA containing CAI and the promoter. This fragment was subsequently cloned into the linearized vector (*Eco*RI) pUC18R6KT-miniTn7T-Km^R^ using the In-Fusion HD Enzyme Premix (Clontech). The resulting plasmid was called pUC18R6KT-miniTn7T-Km^R^_Bs513_CAI (Additional file 2) and was transformed into *E. coli* PIR1 and then to *E. coli* S17 λpir. After, the plasmid was introduced into the different *Brucella* strains by a tetraparental conjugation [42]. The strains were called *B. abortus* 2308W Tn7_Bs513_CAI, *B. abortus* 292 Tn7_Bs513_CAI and *B. abortus* 544 Tn7 _Bs513_CAI. The insertion of the transposon was confirmed by PCR (see above and Additional file 3).

When necessary, constructs without kanamycin resistance cassette were obtained following the protocol set up by Martínez-Gómez et al. [43].

### Growth measurements

The strains were inoculated into 10 mL of TSB or TYSB in a 50 mL flask and incubated at 37 °C for 18 h with or without orbital shaking, in an atmosphere with 5% CO_2_ in the case of CO_2_-dependent strains. Then, these bacteria were harvested by centrifugation, resuspended in 10 mL of the test medium at an optical density at 600 nm (OD_600nm_) of 0.1, and incubated under the same conditions for 18 h. These exponentially growing bacteria were harvested by centrifugation, resuspended at an OD_600nm_ of 0.1 (equivalent to 0.05 readings in the Bioscreen apparatus) in the test medium in appropriate multiwell plates (200 μL/well) and cultivated in a Bioscreen C (Lab Systems) apparatus with continuous shaking at 37 °C. Absorbance values at 420–580 nm were automatically recorded at 30 min-intervals. All experiments were performed in triplicate. Controls with medium and no bacteria were included in all experiments.

### Studies in mice

Seven-week-old female BALB/c mice (Harlan Laboratories; Bicester, United Kingdom) were accommodated in the facilities of “Centro de Investigación y Tecnología Agroalimentaria de Aragón” (CITA; Registration code ES502970012025) for 2 weeks before and during the experiments, with water and food ad libitum under P3 biosafety containment conditions. The animal handling and other procedures were in accordance with the current European (directive 86/609/EEC) and Spanish (RD 53/2013) legislations, supervised by the Animal Welfare Committee of the CITA (2014-20).

To prepare inocula, BABS-grown bacteria were harvested, adjusted spectrophotometrically (OD_600nm_ = 0.170) in sterile buffered saline (BSS; 0.85% NaCl, 0.1% KH_2_PO_4_, 0.2% K_2_HPO_4_; pH 6.85) and diluted in the same diluent up to approximately 5 × 10^7^ CFU/mL. For each bacterial strain, five mice were intraperitoneally inoculated with 0.1 mL/mouse, the exact doses assessed retrospectively by plating dilutions of the inocula. The number of CFU in spleen was determined at 3 and 8 weeks post-inoculation. For this, the spleens were aseptically removed and individually weighed and homogenized in 9 volumes of BSS. Serial tenfold dilutions of each homogenate were performed, and each dilution was plated by triplicate. Plates were incubated at 37 °C, without CO_2_, for 5 days. The identity of the spleen isolates was confirmed by PCR. The individual number of CFU/spleen was normalized by logarithmic transformation, and the mean log CFU/spleen values and the standard deviations (*n* = 5) were calculated. Statistical comparisons were performed by Student’s *t*-test.

## Results

### ORF sequences suggest a critical role of CAII in CO_2_-independence

We first analyzed whether the sequences of _Bs1330_CAI and _Bs1330_CAII, respectively encoded by BRA0788 and BR1829 of *B. suis* 1330 and with proved CA activity, had orthologues in reference and collection strains representative of the CO_2_-independent and -dependent *Brucella* phenotypes (Table 1). This analysis showed that all these brucellae carry _Bs1330_CAI and _Bs1330_CAII orthologues, with the peculiarities summarized below (for further details, see Additional files 4 and 5).

*B. suis* 513 _Bs1330_CAI orthologue differed from _Bs1330_CAI only at position 40 (serine instead of leucine) and carried a CAII identical to _Bs1330_CAII. The *B. abortus* 2308W _Bs1330_CAI orthologue differed from _Bs1330_CAI in that the serine at position 40 and valine in position 76 were both substituted by glycine. Similarly, the _Bs1330_CAII orthologue had an extra amino acid (alanine) at position 114. *B. abortus* 292 and 544, both CO_2_-dependent, contained a _Bs1330_CAI orthologue with the same serine and valine substitutions as strain 2308W, and a cytosine insertion at position 338 of the _Bs1330_CAII orthologue leading to a frameshift affecting almost 50% of the protein. In the *B. ovis* PA _Bs1330_CAI orthologue, a deletion of 24 nucleotides at positions 217–240 results in a protein lacking amino acids 74–81, and the insertion of a guanine at the _Bs1330_CAII orthologue originates a frameshift and a protein defective in the last 40 amino acids. *B. ovis* REO198, which is CO_2_-independent, is identical to *B. ovis* PA with respect to the _Bs1330_CAI orthologue. However, the lack of a guanine in the _Bs1330_CAII orthologue three positions after the 521 position guanine of its *B. ovis* PA counterpart restores the reading frame and should allow synthesis of a protein identical to that of *B. suis* 1330 (Additional files 4 and 5). Altogether, the observations strongly suggest that the CO_2_-dependence of *B. ovis* PA, *B. abortus* 292 and 544 is caused by the lack of an active CAII (activity defined empirically as that allowing growth in a normal atmosphere) and, conversely, that mutations in *B. ovis* CAII could account for the spontaneous emergence of CO_2_-independent strains in at least *B. ovis*. On the other hand, these analyses did not allow inferring the relevance of CAI, which was apparently complete in *B. abortus* and *B. suis*.

### CAII is mutated in spontaneous CO_2_-independent mutants

We examined first the validity of the hypothesis on the relevance of CAII for growth in normal air by comparing the putative CA genes of several spontaneous CO_2_-independent mutants that appeared during routine laboratory manipulations with their parental counterparts (Table 1). For the *B. ovis* PA CO_2_-independent mutant (BoPA-CO_2_^mut^), we observed that while the guanine in the CAII gene causing the above-described frameshift was absent, the CAI gene had not undergone any changes. The CO_2_-independent mutants of three recent *B. abortus* isolates (one biovar 1 and two biovar 3) [44] lacked a guanine at position 340 of the CAII gene that was however present in the CO_2_-dependent parental isolates. Altogether, these results support the starting hypothesis that CAII mutations are involved in the emergence of CO_2_-independent mutants and indirectly suggest that CAI is less relevant in the uptake of CO_2_.

### An active CAI is enough by itself to support CO_2_-independent growth of *B. suis* but not of *B. abortus*

**Figure 1:**
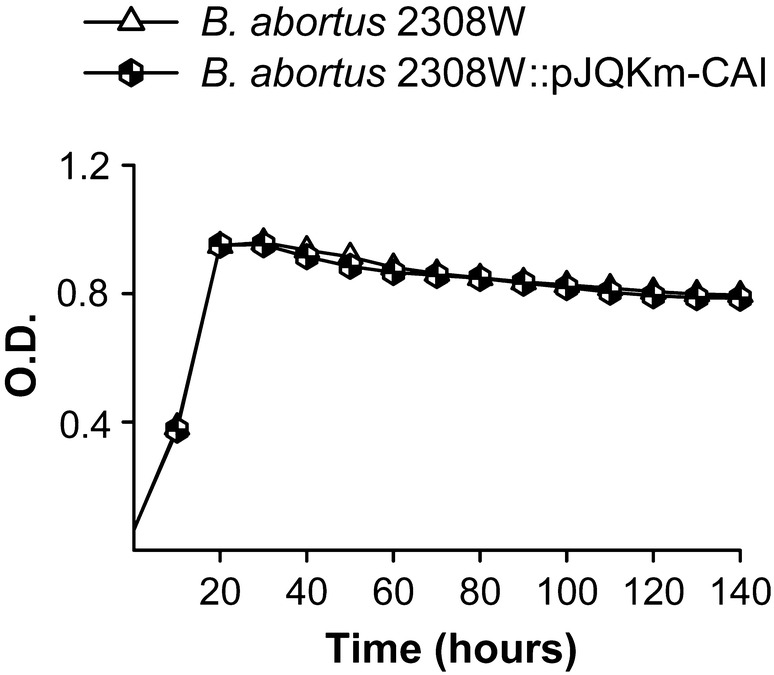
**CAI is dispensable for CO**_**2**_**-independent growth of** ***B. abortus*** **2308W.** Growth of *B. abortus* 2308W and the corresponding insertion mutant in CAI were tested under normal atmospheric conditions in Peptone-Glucose. Each point represents the mean ± standard error (error bars are within the size of the symbols) of technical triplicates. The experiment was repeated three times with similar results.

To compare the physiological importance of CAI and CAII we first carried out mutagenesis in different backgrounds and tested the mutants for CO_2_-independence. Using *B. abortus* 2308W, we found that its CAI mutant (*B. abortus* 2308W::pJQKm-CAI) kept the CO_2_-independent phenotype, proving that CAII by itself can sustain growth under normal atmospheric conditions (Figure 1). In contrast, and despite repeated attempts, we failed to obtain a similar mutant in CAII, suggesting that the *B. abortus* 2308W CAI cannot supply bicarbonate at a rate high enough for growth under normal atmospheric conditions. This is in keeping with the identity of CAI sequence between *B. abortus* 2308W on one hand and *B. abortus* 292 and 544 on the other, and supports the idea that CAI is not active in these three *B. abortus* strains.

**Figure 2:**
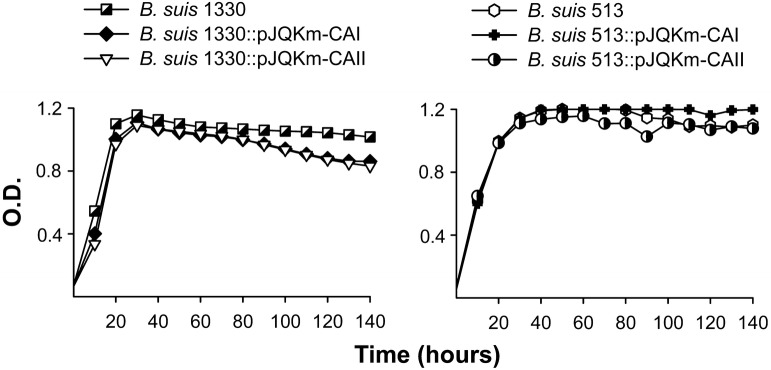
**CAI and CAII are functional in** ***B. suis*** **1330 and 513.** Growth of *B. suis* 1330 and *B. suis* 513 and the corresponding insertion mutants in CAI (*B. suis* 1330::pJQKm-CAI; *B. suis* 513::pJQKm-CAI) and CAII (*B. suis* 1330::pJQKm-CAII; *B. suis* 513::pJQKm-CAII) were tested under normal atmospheric conditions in Peptone-Glucose. Each point represents the mean ± standard error (error bars are within the size of the symbols) of technical triplicates. The experiment was repeated three times with similar results.

Köhler et al. [34] reported recently the failure to obtain *B. suis* 1330 (biovar 1) double CAI–CAII mutants, which together with the analysis of the purified CAI [31–33] strongly suggests that CAI is active in this strain. Thus, we hypothesized that CAI could be inactive in some *Brucella* strains but active in others. Indeed, this possibility was consistent with the observation that the valine in position 76 in *B. suis* 1330 _Bs1330_CAI was substituted by glycine in *B. abortus* 292 and 544 (both CO_2_-dependent) as well as in strain 2308W (see above and Additional file 4) where CAII seemed essential for CO_2_-independence. To validate our hypothesis, we first constructed pJQKm insertion mutants in the _Bs1330_CAI and _Bs1330_CAII genes of *B. suis* 1330 (biovar 1) and found that both were CO_2_-independent (Figure 2). Then, we obtained similar pJQKm insertion mutants in *B. suis* 513 (biovar 5). Again, both mutants kept the CO_2_-independent phenotype of the parental strain, proving that CAI was also active in *B. suis* 513 despite the difference in position 40 (serine instead of leucine) with respect to _Bs1330_CAI (see above and Additional files 4 and 5).

**Figure 3:**
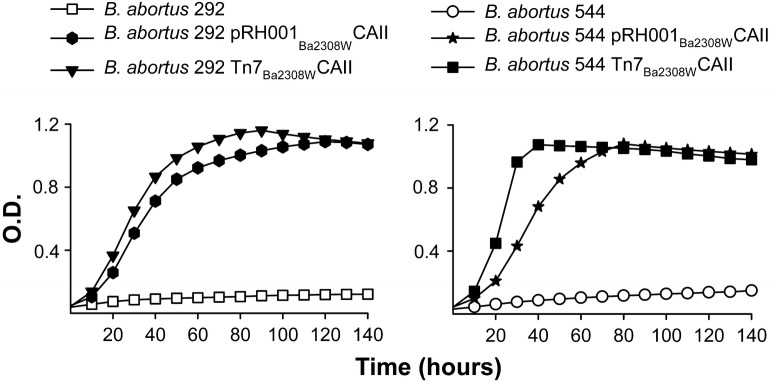
***B. abortus*** **292 and 544 carrying a functional CAII become CO**_**2**_**-independent.** Growth of *B. abortus* strains 292 and 544 and the derivative strains carrying plasmid pRH001_Ba2308W_CAII or a stable Tn7_Ba2308W_CAII insertion in the genome were tested under normal atmospheric conditions in Peptone-Glucose. Each point represents the mean ± standard error (error bars are within the size of the symbols) of technical triplicates. The experiment was repeated three times with similar results.

Once we knew that CAII was essential for CO_2_-independent growth of *B. abortus* 2308 W and that CAI was active in *B. suis* 1330 and 513, we tested whether a functional CAII or CAI could accomplish the same role in CO_2_-dependent *B. abortus* strains. For this, we first constructed a low-copy plasmid (pRH001_Ba2308W_CAII) carrying the gene encoding *B. abortus* 2308W CAII (which we had proven to be active) under the control of its own promoter. When we introduced this plasmid into *B. abortus* 292 and 544 (both CO_2_-dependent), the pRH001_Ba2308W_CAII constructs were able to grow in a normal atmosphere (Figure 3). Then, to circumvent any gene dosage artifacts associated with plasmid constructs, we introduced a miniTn7 carrying _Ba2308W_CAII (Tn7_Ba2308W_CAII) [42] into a neutral site of the genomes of *B. abortus* 292 and 544. We found that, like the strain origin of the CAII gene, the two constructs grew in a normal atmosphere (Figure 3). Then, we did similar experiments with a miniTn7 carrying _Bs513_CAI (which we had proven to be active) and its promoter (Tn7_Bs513_CAI). In this case, however, we found that the *B. abortus* 292 and 544 Tn7_Bs513_CAI constructs failed to grow without CO_2_ enrichment (data not shown) leading to the conclusion that an active CAI was not enough by itself to support CO_2_-independent growth of *B. abortus.*

### The CO_2_-independence mediated by CAI is conditioned by nutrient availability

**Figure 4:**
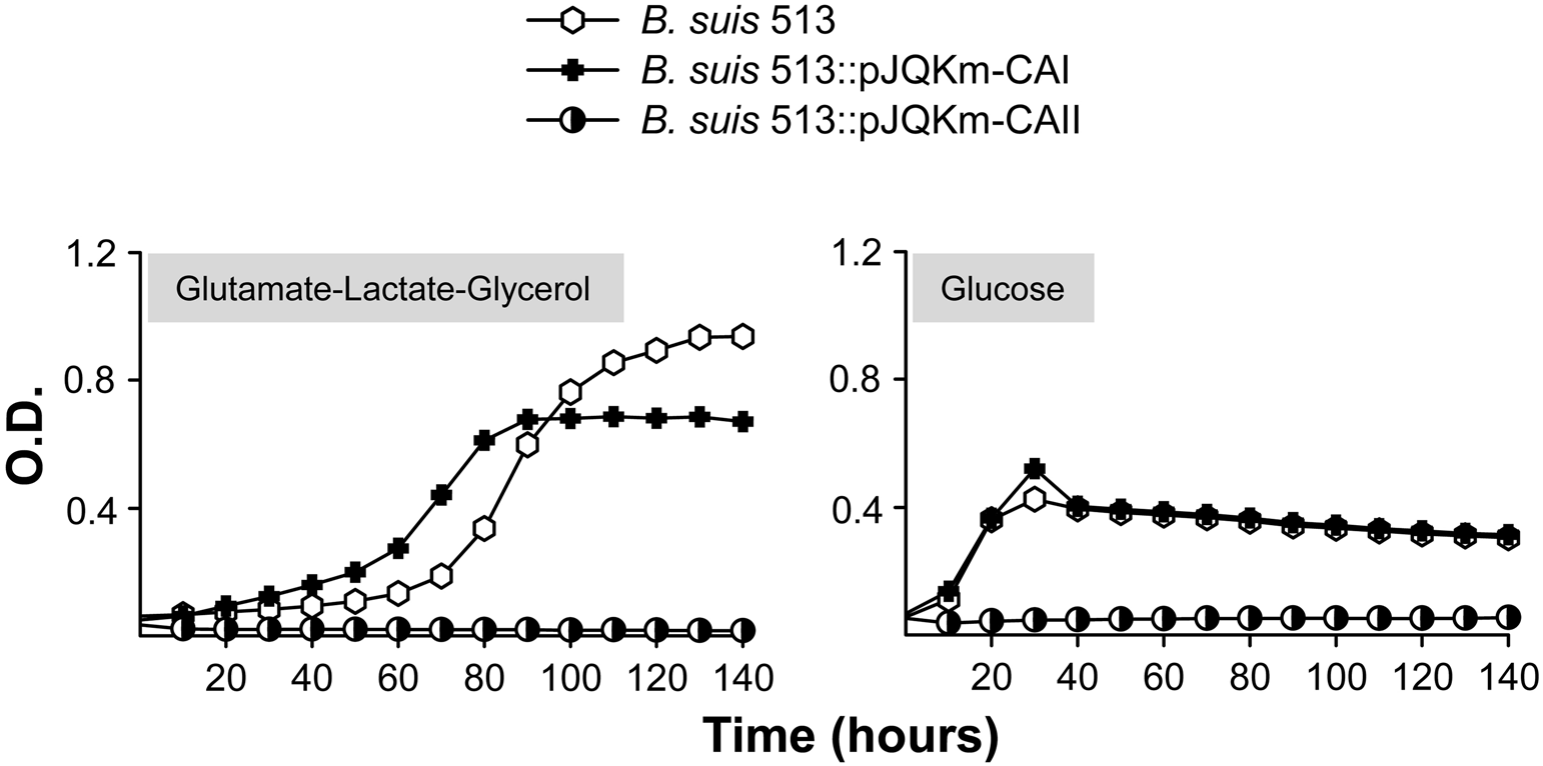
**The CO**_**2**_**-independence mediated by CAI is conditioned by nutrient availability.** Growth of *B. suis* strain 513 and the CA insertion mutants (*B. suis* 513::pJQKm-CAI; *B. suis* 513::pJQKm-CAII) were tested under normal atmospheric conditions in Glutamate–Lactate–Glycerol and Glucose. Each point represents the mean ± standard error (error bars are within the size of the symbols) of technical triplicates. The experiment was repeated three times with similar results.

**Figure 5:**
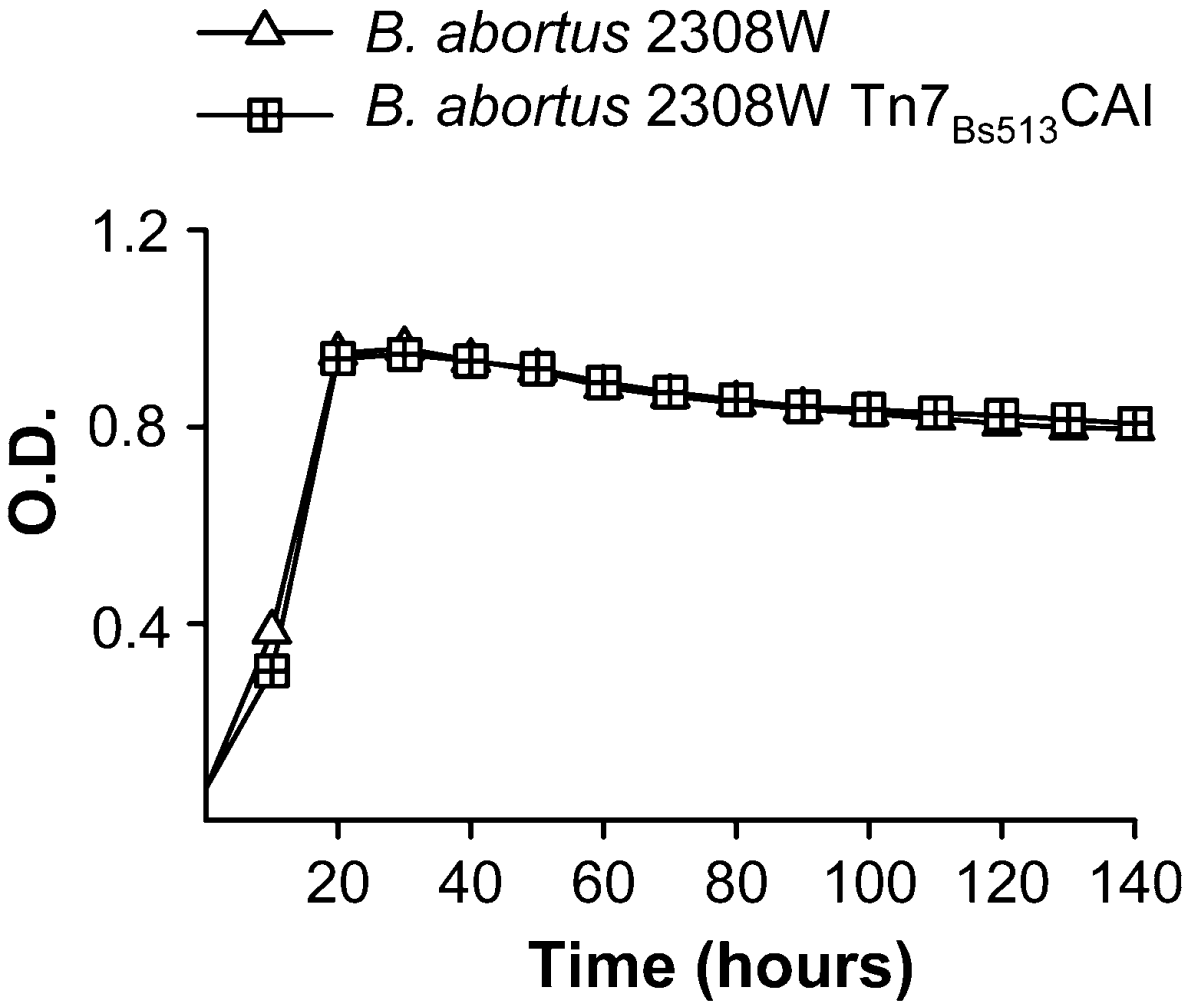
**Insertion of the gene of a functional CAI in** ***B. abortus*** **2308W does not increase growth rates.** Growth of *B. abortus* 2308W and its derivative strain carrying *B. suis* 513 CAI in the genome (*B. abortus* 2308W Tn7_Bs513_CAI) were tested under normal atmospheric conditions in Peptone-Glucose. Each point represents the mean ± standard error (error bars are within the size of the symbols) of technical triplicates. The experiment was repeated three times with similar results.

While the above-described experiments show that an active CAII but not an active CAI was enough to bypass CO_2_-dependence in *B. abortus*, it was not immediately obvious why CAI by itself was enough to support growth of *B. suis* 1330 and *B. suis* 513. However, the *B. suis* CAII (and CAI) mutants were tested for CO_2_-independence in a medium rich in peptones and glucose, conditions that are likely to downplay the role of the anabolic pathways where CA activity is important. Therefore, we reasoned that, depending upon the metabolic abilities of *Brucella* spp. and biovars, experiments in complex media could be not stringent enough to reveal differences between CAI and CAII activities. To analyze this, we took advantage of the almost prototrophic characteristics of *B. suis* 513, a strain that only requires a few vitamins and grows efficiently with limited C supplies [45]. When we tested *B. suis* 513::pJQKm-CAII insertion mutant on Glutamate–Lactate–Glycerol (a gluconeogenic medium [21, 45]) or Glucose as the only C sources, we found that the mutant failed to grow under a normal atmosphere (Figure 4). This result, which shows that *B. suis* 513 CAI cannot meet the biosynthetic demands of this strain in simple media, strongly suggest that CAI is not active enough in less prototrophic species such as *B. abortus* even in complex media and, therefore, that it adds little to the role of CAII. In keeping with this, we found that a *B. abortus* 2308 W construct carrying _Bs513_CAI and its parental strain did not differ in growth rates (Figure 5).

### An active CAII reverts the CO_2_-dependence of *B. ovis* PA and does not alter its multiplication in the mouse model

**Figure 6:**
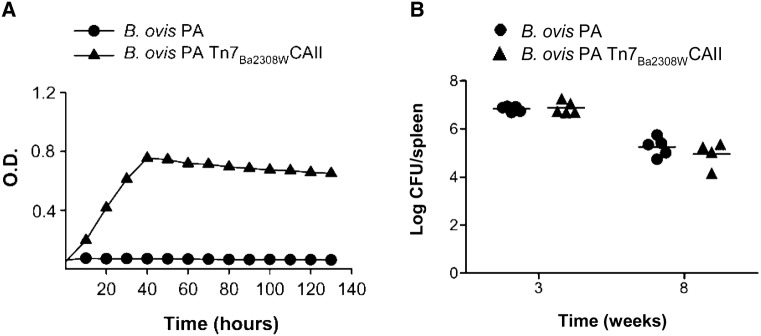
**An active CAII reverts the CO**_**2**_**-dependence of** ***B. ovis*** **PA and does not alter its multiplication in the mouse model. A** Growth of *B. ovis* PA and its derivative strain carrying *B. abortus* 2308W CAII in the genome (*B. ovis* PA Tn7_Ba2308W_CAII) under normal atmospheric conditions in Peptone-Glucose-Yeast Extract-Serum. Each point represents the mean ± standard error (error bars are within the size of the symbols) of technical triplicates. The experiment was repeated three times with similar results. **B** Bacterial loads of *B. ovis* PA and *B. ovis* PA Tn7_Ba2308W_CAII in the spleens of BALB/c mice at 3 and 8 weeks post-infection. No statistical differences were found (Student’s *t*-test).

Among the brucellae, *B. ovis* is notorious for its CO_2-_dependence and fastidious nutritional requirements caused in all likelihood by its comparatively genome degradation [46]. Since the above-described experiments not only proved the chief role of CAII in the *B. abortus* and *B. suis* biovars tested but also provided molecular tools for relieving CO_2_-dependence in *B. abortus*, we introduced Tn7_Ba2308W_CAII into the *B. ovis* PA chromosome and examined the construct for CO_2_-dependence. As can be seen in Figure 6A, *B. ovis* PA Tn7_Ba2308W_CAII grew under normal atmospheric conditions. Then, we used the Tn7_Ba2308W_CAII construct to test whether the CO_2_-dependence and/or this genetic manipulation would alter the virulence of *B. ovis* in the standard mouse model. We found that the introduction of a functional CAII into *B. ovis* PA did not affect the multiplication (acute phase) and permanence (chronicity) of the bacteria in the spleens of BALB/c mouse (Figure 6B).

## Discussion

The metalloenzymes generically designated as CAs catalyze the reversible hydration of CO_2_ into bicarbonate, the substrate of key anaplerotic and biosynthetic enzymes [23]. Many aerobic microorganisms can obtain enough bicarbonate from ambient air (about 0.04% CO_2_) and not surprisingly CA mutants of at least *Ralstonia eutropha*, *Streptococcus pneumoniae*, *Escherichia coli*, *Pseudomonas aeruginosa* and *Corynebacterium glutamicum* are unable to grow under these conditions [25–30]. All these observations made very likely the existence of a relationship between CA deficiencies and the CO_2_-dependence that is characteristic of some *Brucella* biovars and species, a hypothesis proved in this work. If we define CA inactivity as that not high enough to make the bacteria able to grow in a normal atmosphere, we have demonstrated that while *B. suis* 1330 and 513 genomes encode two active CAs (CAI and CAII) only CAII is active in *B. abortus* 2308W and none in representative strains of the CO_2_-dependent *B. abortus* biovars or in *B. ovis* PA. Indeed, the fact that the gene encoding CAII is conserved in *B. abortus* 2308W is in agreement with previous in vitro enzymatic analysis that show that *B. suis* 1330 CAII is a better catalyst for the conversion of CO_2_ to bicarbonate (with an enzymatic activity 1.85 times higher) than _Bs1330_CAI [32].

It has to be stressed that the functional definition of activity used here pertains to the particular physiology of each strain. Although the number of strains tested was necessarily limited, we found evidence supporting the hypothesis that CAI is insufficient to support growth of *Brucella* when the medium is limited to simple C substrates, or even in rich media for those brucellae that display comparatively reduced biosynthetic abilities. Indeed, whereas growth on the minimal media used here requires bicarbonate being incorporated by the reactions catalyzed by enzymes such as phosphoenolpyruvate carboxylase, pyruvate carboxylase, carbamoyl phosphate synthetase, 5-aminoimidazole ribotide carboxylase and enoyl-CoA carboxylases/reductases, growth on rich media does not entail an intense biosynthesis of amino acids and nucleic acid precursors and, therefore, most if not all of the linked pathways should pose no stringent demands for bicarbonate. In the context of this hypothesis, *B. suis*, the fast-growing *B. suis* biovar 5 (strain 513) in particular, on one hand, and *B. ovis,* on the other, would respectively represent two opposite situations. The existence of *Brucella* strains carrying inactivated CAs strongly suggests that this enzymatic activity is not necessary for the persistency in nature of at least *B. abortus* and *B. ovis*. Indeed, the presence of mutations inactivating the metabolic genes may result from the absence of a positive selective pressure, reflecting an adaptation during which the cognate functions become dispensable because of the nutritional environment. Such a *Brucella* adaptation would not be a novelty in intracellular parasites because, while a majority of the genome-sequenced *Proteobacteria* retain a CA gene, intracellular genera such as *Buchnera* and *Rickettsia* contain CA-defective representatives [47]. It remains to be investigated whether such a CA dispensability represents a high CO_2_ tension in their niche, as described for *Symbiobacterium thermophilum* [48], the exploitation of host CAs or the presence of nutrients bypassing metabolic steps connected to CA activity.

It is important to highlight the practical implications of this work. Unraveling the genetic background of *Brucella* CO_2_-dependence allowed us to construct a *B. ovis* CO_2_-independent mutant with practical implications on the diagnosis and control of *B. ovis* infection. Because of the non-zoonotic nature of *B. ovis*, this disease may not always deserve the attention of official programs and it is often overlooked. Control and eventual eradication of *B. melitensis* brucellosis of small ruminants is based on the use of diagnostic tests detecting antibodies to the S-LPS [49] and vaccination with *B. melitensis* Rev 1, a vaccine that also protects against *B. ovis*. However, Rev 1 may interfere in serological diagnosis [49] and it is virulent for humans [50] and resistant to streptomycin (an antibiotic of choice to treat human brucellosis). Owing to these drawbacks, Rev 1 vaccination is discontinued and finally banned in those regions or countries where *B. melitensis* prevalence is considered low enough to implement an exclusively test and slaughter strategy. Withdrawal of Rev 1 vaccination leaves animals unprotected against *B. ovis*, thus favoring the emergence of the disease in areas where *B. melitensis* is almost or totally eradicated. Moreover, *B. ovis* has remained endemic in many areas where *B. melitensis* is not present and Rev 1 vaccination was never implemented [51, 52]. Accordingly, research on *B. ovis*-specific vaccines is an area of increasing interest as these vaccines would neither pose risk of zoonotic infection nor interfere in those *B. melitensis* serological tests detecting S-LPS *O*-polysaccharide antibodies (i.e., rose bengal and complement fixation tests) [53–56]. The CO_2_ requirement represents a significant obstacle in the development of a *B. ovis* live attenuated vaccine for large-scale production. We have demonstrated that the *B. ovis* PA Tn7_Ba2308W_CAII described here not only grows under normal atmospheric conditions but also retains the virulence in at least the accepted laboratory model, thus representing an appropriate tool for the development of such CO_2_-independent attenuated vaccines. For instance, *B. ovis* PA Tn7_Ba2308W_CAII can be used as the background to apply the strategy proposed by Conde-Álvarez et al. [57] based on the deletion of LPS core glycosyltransferases that results in a truncated structure that by uncovering innate immunity targets triggers a potent protective Th1 response. In fact, Soler-Lloréns et al. [56] recently demonstrated that deletion of two of such glycosyltransferases in *B. ovis* PA results in attenuation and suitable vaccine properties in the mouse model. Similarly, the *B. ovis* PA Tn7_Ba2308W_CAII construct could be used to produce the R-specific antigen currently used in *B. ovis* serological tests. This antigen is made of vesicles rich in outer membrane proteins and R-LPS, and both types of components have been shown to be important for optimal sensitivity [58, 59]. Currently, this R antigen is obtained from *B. ovis* REO198 taking advantage of the unusual CO_2_-independence of this strain (Table 1). Yet, *B. ovis* REO198 LPS carries a core oligosaccharide defect that damages the diagnostic epitopes of the R LPS and it is thus likely to yield suboptimal results in serodiagnosis [60]. If this is confirmed, it could be advantageously replaced by *B. ovis* PA Tn7_Ba2308W_CAII. Research is in progress to evaluate the attenuation and protection against *B. ovis* of *B. ovis* PA Tn7_Ba2308W_CAII core glycosyltransferase mutants as well as the diagnostic properties of R antigens obtained from this strain. In summary, the evidence presented in this work not only clarifies the biochemical basis of an important *Brucella* phenotype but also provides a tool for large-scale production of *B. ovis* diagnostic antigens and vaccines.

## Additional files

**Additional file 1.** **Insertion mutants and genetic constructs obtained in this work.**

**Additional file 2.** ***E. coli*** **strains and plasmids.**

**Additional file 3.** **Primers.**

**Additional file 4.** **Structure-based sequence alignment of CAI.** Gear symbols denote the residues observed as zinc ligands. The secondary structural features are indicated above the alignment (helices indicated as cylinders, strands as arrows). In bold, the six amino acid-sequence conserved in both CAI and CAII. Underlined, the glycine that has substituted the valine that is present in the *B. suis* strains.

**Additional file 5.** **Structure-based sequence alignment of CAII.** Gear symbols denote the residues observed as zinc ligands. The secondary structural features are indicated above the alignment (helices indicated as cylinders, strands as arrows). In bold the six amino acid-sequence conserved in both CAI and CAII.
