# Supplementary material for: The CO2-dependence of Brucella ovis and Brucella abortus biovars is caused by defective carbonic anhydrases
Source: Vet Res. 2018 Sep 5;49:85. doi: 10.1186/s13567-018-0583-1 (PMC6126018; doi:10.1186/s13567-018-0583-1)
Supplement: Supplementary file 1 — Additional file 1. Insertion mutants and genetic constructs obtained in this work. [file 13567_2018_583_MOESM1_ESM.pdf]

Strain	Characteristics	Code
Brucella abortus		
B. abortus 292 derivatives		
B. abortus 292 pRH001$_{Ba2308W}$CAII	*B. abortus* 292 carrying pRH001$_{Ba2308W}$CAII	AZB179
B. abortus 292 Tn7$_{Ba2308W}$CAII	*B. abortus* 292 carrying Tn7$_{Ba2308W}$CAII	AZB214
B. abortus 292 Tn7$_{Bs513}$CAI	*B. abortus* 292 carrying Tn7$_{Bs513}$CAI	AZB345
B. abortus 544 derivatives		
B. abortus 544 pRH001$_{Ba2308W}$CAII	*B. abortus* 544 carrying pRH001$_{Ba2308W}$CAII	AZB178
B. abortus 544 Tn7$_{Ba2308W}$CAII	*B. abortus* 544 carrying Tn7$_{Ba2308W}$ CAII	AZB204
B. abortus 544 Tn7$_{Bs513}$CAI	*B. abortus* 544 carrying Tn7$_{Bs513}$CAI	AZB344
B. abortus 2308W derivatives		
B. abortus 2308W::pJQKm-CAI	*B. abortus* 2308W insertion mutant in CAI	AZB180
B. abortus 2308W Tn7$_{Bs513}$CAI	*B. abortus* 2308W carrying Tn7$_{Bs513}$CAI	AZB337
Brucella suis		
B. suis 1330 derivatives		
B. suis 1330::pJQKm-CAI	*B. suis* 1330 insertion mutant in CAI	AZB332
B. suis 1330::pJQKm-CAII	*B. suis* 1330 insertion mutant in CAII	AZB333
B. suis 513 derivatives		
B. suis 513::pJQKm-CAI	*B. suis* 513 insertion mutant in CAI	AZB334
B. suis 513::pJQKm-CAII	*B. suis* 513 insertion mutant in CAII	AZB335
Brucella ovis		
B. ovis PA derivatives		
B. ovis PA Tn7$_{Ba2308W}$CAII	*B. ovis* PA carrying Tn7$_{Ba2308W}$ CAII	AZB248
