# Supplementary material for: The CO2-dependence of Brucella ovis and Brucella abortus biovars is caused by defective carbonic anhydrases
Source: Vet Res. 2018 Sep 5;49:85. doi: 10.1186/s13567-018-0583-1 (PMC6126018; doi:10.1186/s13567-018-0583-1)
Supplement: Supplementary file 2 — Additional file 2. E. coli strains and plasmids. [file 13567_2018_583_MOESM2_ESM.pdf]

Strain/Vector or plasmid	Characteristics	Reference/Source
***E. coli* strain**		
TOP10	F– mcrA Δ(mrr-hsdRMS-mcrBC) Φ80lacZΔM15 ΔlacX74 recA1 araD139 Δ(ara leu) 7697 galU galK rpsL(StrR) endA1 nupG λ-	Invitrogen
DH5α	F- Φ80lacZΔM15 Δ(lacZYA-argF) U169 recA1 end A1 hsdR17(rk-, mk+) phoA supE44 thi-1 gyrA96 relA1 λ-	Invitrogen
Stellar	F–, endA1, supE44, thi-1, recA1, relA1, gyrA96 phoA, Φ80d lacZΔ M15, Δ(lacZYA- argF) U169 Δ(mrr-hsdRMS-mcrBC), ΔmcrA, λ–	Clontech
S17 λpir	Mating strain with plasmid RP4 inserted into the chromosome (Tpr Smr recA thi hsdRM+, lambda pir phage lysogen RP4::2-Tc::Mu::Km Tn7)	[38,39]
PIR1	F- Δlac169 rpoS(Am) robA1 creC510 hsdR514 endA recA1 uidA(ΔMluI)::pir-116	Invitrogen
SM10 λpir	thi-1, thr, leu, tonA, lacY, supE, recA::RP4-2-Tc::Mu, λ*pir*.	[39]
HB101	F- mcrB mrr hsdS20(rB- mB-) recA13 leuB6 ara-14 proA2 lacY1 galK2 xyl-5 mtl-1 rpsL20(SmR) glnV44 λ-	[42]
β2150	thrB1004 pro thi strA hsdS lacZDM15 (F' lacZDM15 laclq traD36 proA+ proB+) DdapA::erm (Ermr) pir	[43]
**Vector or plasmid**		
pDONOR223	Cloning vector for Gateway technology	Invitrogen
$pDONOR223_{Ba2308W}CAII$	*B. abortus* 2308W chromosomal DNA containing the complete ORF BAW_11758 (CAII; identical sequence to that of BAB1_1837) with its promoter generated by PCR and cloned into pDONOR223	This work
pRH001	Derivate of pMR10, Km^R, Cm^R	[40]
$pRH001_{Ba2308W}CAII$	attL1-attL2 fragment of pDONOR223-$CAII_{ba2308W}$ cloned into the attR1-attR2 sites of pRH001	This work
pCR2.1-TOPO®	Cloning vector, Km^R	Invitrogen
$pCR2.1_{Ba2308W}CAI$	302 bp of *B. abortus* 2308W chromosomal DNA containing an internal fragment of BAW_20431 (CAI; identical sequence to that of BAB2_0449) generated by PCR and cloned into pCR2.1	This work
$pCR2.1_{Ba2308W}CAII$	323 bp of *B. abortus* 2308 chromosomal DNA containing an internal fragment of BAW_11758 (CAII; identical sequence to that of BAB1_1837) generated by PCR and cloned into pCR2.1	This work
pJQKm	Derivate of pJQ200KS+, Km^R, Gm^S, suicide vector	[37]
$pJQKm_{Ba2308W}CAI$	*BamH*I/*Xba*I fragment from pCR2.1-$CAI_{ba2308W}$ cloned into the corresponding site of pJQKm	This work
$pJQKm_{Ba2308W}CAII$	*BamH*I/*Xba*I fragment from pCR2.1-$CAII_{ba2308W}$ cloned into the corresponding site of pJQKm	This work
pUC18R6KT-miniTn7-Km^R	Broad host-range mini-Tn7 vector	[41]
pUC18R6KT-miniTn7-$Km^R{}_{Ba2308W}CAII$	966 bp of *B. abortus* 2308W chromosomal DNA containing the ORF BAW_11758 (CAII; identical sequence to that of BAB1_1837) and its promoter generated by PCR and cloned into pUC18R6KT-miniTn7-Km^R	This work
pUC18R6KT-miniTn7-$Km^R{}_{Bs513}CAI$	987 bp of *B. suis* 513 chromosomal DNA containing the BAW_20431 ortholog ORF and its promotor generated by PCR and cloned into pUC18R6KT-miniTn7-Km^R	This work
pTNS2	Plasmid expressing *tnsABCD* from p*lac*, Amp^R	[42]
pRK2013	Helper vector containing genes *tra* and *mob*, Km^R	[42]
pNPTS138Cm	Suicide vector, Cm^R	Addgene
pNPTS138CmΔkm (pRCI-65)	*EcoR*I fragment containing a deleted kanamycin resistance gene cloned in the corresponding site of pNPTS138Cm	[43]
