# Supplementary material for: The CO2-dependence of Brucella ovis and Brucella abortus biovars is caused by defective carbonic anhydrases
Source: Vet Res. 2018 Sep 5;49:85. doi: 10.1186/s13567-018-0583-1 (PMC6126018; doi:10.1186/s13567-018-0583-1)
Supplement: Supplementary file 3 — Additional file 3. Primers. [file 13567_2018_583_MOESM3_ESM.pdf]

Primers	Sequence 5'-3'[1]	Comments	Tm (ºC)
CAII-Fw-Gw	GGGGACAAGTTTGTACAAAAAAGCAGGCTTCCGCTGCCGTGTTTGAAATCA	Used to construct pDONOR223$_{Ba2308W}$CAII	60
CAII-Rv-Gw	GGGGACCACTTTGTACAAGAAAGCTGGGTCTCAAAGTTCAGGGCGTTTGAA	Used to construct pDONOR223$_{Ba2308W}$CAII	60
CAI-F1-Ins	GAATTTCTATGGATCGGCTGTT	Used to construct pCR2.1$_{Ba2308W}$CAI	60
CAI-R2-Ins	CGGTCCTGCGTGTTTTCTAT	Used to construct pCR2.1$_{Ba2308W}$CAI	60
CAII-F1-Ins	CAATGTGGCCAATCTCATTC	Used to construct pCR2.1$_{Ba2308W}$CAII	60
CAII-R2-Ins	GCGAATAGCGGATCGAAATA	Used to construct pCR2.1$_{Ba2308W}$CAII	60
CAII-IF-F1	***CCGGGCTGCAGGAATT***CGCTGCCGTGTTTGAAATCA	Used to construct pUC18R6KT-miniTn7-Km$^{R}_{Ba2308W}$CAII	60
CAII-IF-R2	***AGCTTCTCGAGGAATT***TCAAAGTTCAGGGCGTTTGAA	Used to construct pUC18R6KT-miniTn7-Km$^{R}_{Ba2308W}$CAII	60
CAI-IF-F1	***CCGGGCTGCAGGAATT***TGTGGAATTGCACCGACAC	Used to construct pUC18R6KT-miniTn7-Km$^{R}_{Bs513}$CAI	60
CAI-IF-R2	***AGCTTCTCGAGGAATT***CAATTATTCTGCCGGTTGG	Used to construct pUC18R6KT-miniTn7-Km$^{R}_{Bs513}$CAI	60
CAI-F1-Sec	CCATGGCATCATCGACAA	Used to sequence CAI	60
CAII-F1-Sec	GCTTTCACCGAGCGATTTTA	Used to sequence CAII	60
CAI-Fw	TGTGGAATTGCACCGACAC	Used to confirm recombination in CAI insertion mutants and to amplify CAI in the spontaneous mutants	58
CAI-Rv	TAATTCTGCCGGTTGGCAG	Used to confirm recombination in CAI insertion mutants and to amplify CAI in the spontaneous mutants	58
CAII-Fw	CGCTGCCGTGTTTGAAATCA	Used to confirm recombination in CAII insertion mutants and to amplify CAII in the spontaneous mutants	60
CAII-Rv	TCAAAGTTCAGGGCGTTTGAA	Used to confirm recombination in CAII insertion mutants and to amplify CAII in the spontaneous mutants	60
M13Fw	GTAAAACGACGGCCAG	Used to confirm recombination in insertion mutants	50
M13Rv	CAGGAAACAGCTATGAC	Used to confirm recombination in insertion mutants	50
Tn7F	TGGCTAAAGCAAACTCTTCATTT	Used to confirm the insertion of the transposon	59
Tn7R	GCGGATTTGTCCTACTCAGG	Used to confirm the insertion of the transposon	59
Gmls_B	GTCCTTATGGGAACGGACGT	Used to confirm the insertion of the transposon	59
RecG	TATATTCTGGCGAGCGATCC	Used to confirm the insertion of the transposon	59
Ptn7-L	ATTAGCTTACGACGCTACACCC	Used to confirm the insertion of the transposon	59
Ptn7-R	CACAGCATAACTGGACTGATT	Used to confirm the insertion of the transposon	59

[1]Underlined, attB adapters for cloning using the Gateway® cloning system (Invitrogen). In bold and italics, adapters for cloning using the In-Fusion® HD Cloning Kit (Takara).
