# Supplementary material for: The CO2-dependence of Brucella ovis and Brucella abortus biovars is caused by defective carbonic anhydrases
Source: Vet Res. 2018 Sep 5;49:85. doi: 10.1186/s13567-018-0583-1 (PMC6126018; doi:10.1186/s13567-018-0583-1)
Supplement: Supplementary file 4 — Additional file 4. Structure-based sequence alignment of CAI. Gear symbols denote the residues observed as zinc ligands. The secondary structural features are indicated above the alignment (helices indicated as cylinders, strands as arrows). In bold, the six amino acid-sequence conserved in both CAI and CAII. Underlined, the glycine that has substituted the valine that is present in the B. suis strains. [file 13567_2018_583_MOESM4_ESM.pdf]

```
Bs1330      MPMKNDHSPDQRTLSELFEHNRQWAAEKQEKDPEYFSRLLSSQRPEFLWIGCSDSRVPANVVTGLQPGEVFVH   73
Bs513       MPMKNDHSPDQRTLSELFEHNRQWAAEKQEKDPEYFSRLSSSQRPEFLWIGCSDSRVPANVVTGLQPGEVFVH
Ba2308W     MPMKNDHSPDQRTLSELFEHNRQWAAEKQEKDPEYFSRLSSSQRPEFLWIGCSDSRVPANVVTGLQPGEVFVH
Ba292       MPMKNDHSPDQRTLSELFEHNRQWAAEKQEKDPEYFSRLSSSQRPEFLWIGCSDSRVPANVVTGLQPGEVFVH
Ba544       MPMKNDHSPDQRTLSELFEHNRQWAAEKQEKDPEYFSRLSSSQRPEFLWIGCSDSRVPANVVTGLQPGEVFVH
BoPA        MPMKNDHSPDQRTLSELFEHNRQWAAEKQEKDPEYFSRLSSSQRPEFLWIGCSDSRVPANVVTGLQPGEVFVH
BoREO       MPMKNDHSPDQRTLSELFEHNRQWAAEKQEKDPEYFSRLSSSQRPEFLWIGCSDSRVPANVVTGLQPGEVFVH

Bs1330      RNVANLVHRADLNLLSVLEFAVGVLEIKHIIVCGHYGCGGVRAAMDGYGHGIIDNWLQPIRDIAQANQAELDT   146
Bs513       RNVANLVHRADLNLLSVLEFAVGVLEIKHIIVCGHYGCGGVRAAMDGYGHGIIDNWLQPIRDIAQANQAELDT
Ba2308W     RNGANLVHRADLNLLSVLEFAVGVLEIKHIIVCGHYGCGGVRAAMDGYGHGIIDNWLQPIRDIAQANQAELDT
Ba292       RNGANLVHRADLNLLSVLEFAVGVLEIKHIIVCGHYGCGGVRAAMDGYGHGIIDNWLQPIRDIAQANQAELDT
Ba544       RNGANLVHRADLNLLSVLEFAVGVLEIKHIIVCGHYGCGGVRAAMDGYGHGIIDNWLQPIRDIAQANQAELDT
BoPA        --------RADLNLLSVLEFAVGVLEIKHIIVCGHYGCGGVRAAMDGYGHGIIDNWLQPIRDIAQANQAELDT
BoREO       --------RADLNLLSVLEFAVGVLEIKHIIVCGHYGCGGVRAAMDGYGHGIIDNWLQPIRDIAQANQAELDT

Bs1330      IENTQDRLDRLCELSVSSQVESLSRTPVLQSAWKDGKDIIVHGWMYNLKDGLLRDIGCDCTRNALQFACQPAE   219
Bs513       IENTQDRLDRLCELSVSSQVESLSRTPVLQSAWKDGKDIIVHGWMYNLKDGLLRDIGCDCTRNALQFACQPAE
Ba2308W     IENTQDRLDRLCELSVSSQVESLSRTPVLQSAWKDGKDIIVHGWMYNLKDGLLRDIGCDCTRNALQFACQPAE
Ba292       IENTQDRLDRLCELSVSSQVESLSRTPVLQSAWKDGKDIIVHGWMYNLKDGLLRDIGCDCTRNALQFACQPAE
Ba544       IENTQDRLDRLCELSVSSQVESLSRTPVLQSAWKDGKDIIVHGWMYNLKDGLLRDIGCDCTRNALQFACQPAE
BoPA        IENTQDRLDRLCELSVSSQVESLSRTPVLQSAWKDGKDIIVHGWMYNLKDGLLRDIGCDCTRNALQFACQPAE
BoREO       IENTQDRLDRLCELSVSSQVESLSRTPVLQSAWKDGKDIIVHGWMYNLKDGLLRDIGCDCTRNALQFACQPAE
```
