# Supplementary material for: The CO2-dependence of Brucella ovis and Brucella abortus biovars is caused by defective carbonic anhydrases
Source: Vet Res. 2018 Sep 5;49:85. doi: 10.1186/s13567-018-0583-1 (PMC6126018; doi:10.1186/s13567-018-0583-1)
Supplement: Supplementary file 5 — Additional file 5. Structure-based sequence alignment of CAII. Gear symbols denote the residues observed as zinc ligands. The secondary structural features are indicated above the alignment (helices indicated as cylinders, strands as arrows). In bold the six amino acid-sequence conserved in both CAI and CAII. [file 13567_2018_583_MOESM5_ESM.pdf]

```
Bs1330      MADLPDSLLAGYKTFMSEHFAHETARYRDLAEKGQSPETLVVACCDSRAAPETIFNAAPGEIFVLRNVANLIP   73
Bs513       MADLPDSLLAGYKTFMSEHFAHETARYRDLAEKGQSPETLVVACCDSRAAPETIFNAAPGEIFVLRNVANLIP
Ba2308W     MADLPDSLLAGYKTFMSEHFAHETARYRDLAEKGQSPETLVVACCDSRAAPETIFNAAPGEIFVLRNVANLIP
Ba292       MADLPDSLLAGYKTFMSEHFAHETARYRDLAEKGQSPETLVVACCDSRAAPETIFNAAPGEIFVLRNVANLIP
Ba544       MADLPDSLLAGYKTFMSEHFAHETARYRDLAEKGQSPETLVVACCDSRAAPETIFNAAPGEIFVLRNVANLIP
BoPA        MADLPDSLLAGYKTFMSEHFAHETARYRDLAEKGQSPETLVVACCDSRAAPETIFNAAPGEIFVLRNVANLIP
BoREO       MADLPDSLLAGYKTFMSEHFAHETARYRDLAEKGQSPETLVVACCDSRAAPETIFNAAPGEIFVLRNVANLIP

Bs1330      PYEPDGEYHAASAALEFAVQSLKVKHIVVMGHGRCGGIKA-ALDTESAPLSPSDFIGKWMSLISPAAEAISGN   145
Bs513       PYEPDGEYHAASAALEFAVQSLKVKHIVVMGHGRCGGIKA-ALDTESAPLSPSDFIGKWMSLISPAAEAISGN
Ba2308W     PYEPDGEYHAASAALEFAVQSLKVKHIVVMGHGRCGGIKAAALDTESAPLSPSDFIGKWMSLISPAAEAISGN
Ba292       PYEPDGEYHAASAALEFAVQSLKVKHIVVMGHGRCGGIKAGARH-----------------------------
Ba544       PYEPDGEYHAASAALEFAVQSLKVKHIVVMGHGRCGGIKAGARH-----------------------------
BoPA        PYEPDGEYHAASAALEFAVQSLKVKHIVVMGHGRCGGIKA-ALDTESAPLSPSDFIGKWMSLISPAAEAISGN
BoREO       PYEPDGEYHAASAALEFAVQSLKVKHIVVMGHGRCGGIKA-ALDTESAPLSPSDFIGKWMSLISPAAEAISGN

Bs1330      ALMTQSERHTALERISIRYSLANLRTFPCVDILEKKGKLTLHGAWFDISTGELWVMDHRTGDFKRPEL        213
Bs513       ALMTQSERHTALERISIRYSLANLRTFPCVDILEKKGKLTLHGAWFDISTGELWVMDHQTGDFKRPEL
Ba2308W     ALMTQSERHTALERISIRYSLANLRTFPCVDILEKKGKLTLHGAWFDISTGELWVMDHQTGDFKRPEL
Ba292       --------------------------------------------------------------------
Ba544       --------------------------------------------------------------------
BoPA        ALMTQSERHTALERISIRYSLANLRTFPW---------------------------------------
BoREO       ALMTQSERHTALERISIRYSLANLRTFPWLDILEKKGKLTLHGAWFDISTGELWVMDHQTGDFKRPEL
```
